# Engagement as a Driver of Growth of Online Health Forums: Observational Study

**DOI:** 10.2196/jmir.7249

**Published:** 2017-08-29

**Authors:** Rahul Gopalsamy, Alexander Semenov, Eduardo Pasiliao, Scott McIntosh, Alexander Nikolaev

**Affiliations:** ^1^ Social Optimization Laboratory Department of Industrial and Systems Engineering University at Buffalo Buffalo, NY United States; ^2^ SOMEA Group Faculty of Information Technology University of Jyväskylä Jyväskylä Finland; ^3^ Munitions Directorate Air Force Research Laboratory Eglin, FL United States; ^4^ Department of Public Health Sciences University of Rochester Rochester, NY United States

**Keywords:** online health forum, online health community, engagement, engagement capacity, superuser, eHealth, social network analysis

## Abstract

**Background:**

The emerging research on nurturing the growth of online communities posits that it is in part attributed to network effects, wherein every increase in the volume of user-generated content increases the value of the community in the eyes of its potential new members. The recently introduced metric *engagement capacity* offers a means of quantitatively assessing the ability of online platform users to engage each other into generating content; meanwhile, the quantity *engagement value* is useful for quantifying communication-based platform use. If the claim that higher engagement leads to accelerated growth holds true for online health forums (OHFs), then engagement tracking should become an important tool in the arsenal of OHF managers. Indeed, it might allow for quantifying the ability of an OHF to exploit network effects, thus predicting the OHF’s future success.

**Objective:**

This study aimed to empirically analyze the relationship between internal OHF use (quantified using *engagement measurement)*, and external growth.

**Methods:**

We collected data from 7 OHFs posted between the years 1999 and 2016. Longitudinal analyses were conducted by evaluating engagement in the OHFs over time. We analyzed 2-way causality effects between the engagement value and metrics evaluating OHF growth using Granger causality tests. User activity metrics per week were correlated with engagement metrics, followed by linear regression analyses.

**Results:**

Observational data showed a 1-way causal relationship between the OHF engagement value and reach (*P*=.02). We detected a 2-way causal relationship between the engagement value and delurking, with further analysis indicating that the engagement value was more likely to cause delurking (*P*<.001 with lag 2; for the reverse hypothesis, *P*=.01 with lag 2). Users who engaged each other more were more likely (up to 14 times, depending on how much one user engaged another) to develop personal connections. Finally, we found that the more engaging an OHF user was in a given week, the more likely (up to 2 times, depending on their ability to engage others) they were to remain active in the OHF in the following week.

**Conclusions:**

This study supports the claim that network effects play an important role in accelerating OHF growth, opening the door to exploiting these effects in calculated ways. In such efforts, engagement metrics can be used to monitor the “health” of an OHF and to identify the users most important to its success.

## Introduction

### Background

Online health forums (OHFs) enable computer-mediated communication on health and health-related issues through the Internet. People use OHFs to seek emotional support, exchange information, ask for help, or simply become part of a community [1]. When the members of an OHF communicate with each other in forum threads, they generate content, which generally benefits other members. For example, members may provide emotional support to each other by sharing personal experiences and stories that may make peers open up in turn. Despite the benefits of OHFs [2], many of them fail due to low user activity. It has been observed that, typically, only 1% of OHF users generate most of the OHF content; meanwhile, 90% of the users are observers, also called lurkers, who rarely leave traces of participation [3].

A growing body of literature addresses the issues of sustaining user activity in online communities. To this end, researchers have used social psychology theories to inform the design of platforms [4,5]. The underlying theme of this line of research is to understand the mechanism of human motivation for the creation of public content. The different branches of this research explore, for example, the uniqueness and role of each user’s contributions in accomplishing group goals [6] and user attachment to a group [7] as motivating factors to keep contributing to the group. Another large-scale empirical investigation distilled the identity formation principles in online communities [8]. These studies, among others, concur that psychological factors are important drivers behind the dynamics of growth of online user-generated content.

Early quantitative analyses of health-related online communication employed such aggregate metrics as the number of registered users, number of contributed posts per user, and log-in frequency [9,10]. Individual characteristics, such as age, sex, location, and time of joining the platform, have been used to complement activity-based observations in the search for trends in online user activity. More recently, researchers have employed the methods and tools from social network analysis to quantify pairwise user interaction. The nodes in a social network, constructed based on online platform activity data, represent the users of the platform, while the ties (edges) represent the relationships between the users: for example, 2 users may share a tie if they have often posted messages within the same threads or have labeled each other as friends using the platform’s interface. The centrality metrics and the structure of the resulting network reveal most well-connected users and subcommunities, the knowledge of which may be informative about the evolution of the platform’s user base [11-13]. Note that all the above-mentioned metrics are good for descriptive purposes; however, they have not been successfully used for predicting OHF future activity.

### The Network Effect Hypothesis in Conjunction With Online Health Forum Growth

Positive network effect is a phenomenon initially studied in economics [14]. It explains the mechanism behind the process where the value of goods or services tends to increase as more consumers begin to use them: for example, a sale of each additional unit of goods may increase the value of the goods through positive *network effects*, also referred to as *network externalities*. On an online platform, such effects may occur when its users author new content [15]. This proposition has motivated attempts to quantitatively describe the role that network externalities may play in the growth of online communities. The key premise here is that OHFs grow through their existing users: every contributed post (that is responded to) fosters user “bonding” and enhances the positive network externalities. Therefore, by identifying, encouraging, and perhaps incentivizing the most engaging content contributors, OHF managers could become more successful at keeping their prohealth online forums growing, achieving a greater impact on people’s health [16].

### Prior Work

This study used a method of measuring engagement in OHF communication based on the recently introduced theoretical work on quantifying online users’ ability to engage peers in conversations. To this end, Nikolaev et al [17] recently introduced the terms *engagement value* and *engagement capacity*, with cooperative game theory employed for measuring the latter. In particular, they offered a means to quantify users’ ability to engage peers. The details of the engagement quantification are provided in the Methods section, accompanied by an illustrative example. They also presented *reach*, introduced within the RE-AIM (reach, efficacy, adoption, implementation, maintenance) program evaluation framework [18], as the key dimension of impact of an online platform; the main premise of their work is that internal growth of a platform—that is, its use that can be quantified through engagement measurement—is responsible for its reach (external growth). However, so far, the latter claim has been presented only as an application-independent proposition, albeit intuitively justifiable. An empirical validation of this proposition, in particular in application to OHFs, would put the research of engagement on more solid ground, simultaneously establishing its practical value.

### Objective

The objective of this study was to analyze the relationship between engagement and OHF growth over time, relying on longitudinal data of real-world OHFs. To this end, we formulated 4 research questions, motivated on the one hand by observations of OHF activity, reported in the existing research literature, and on the other hand by the logic behind the utility of engagement measurement.

An OHF full of conversations is more appealing to external readers, compared with one with little content or lots of messages to which no one has responded. Successful and voluminous conversational engagement can be expected to fuel both the higher intensity of user interaction on an OHF and the word-of-mouth effect, with the latter positively affecting the number of people who become aware of the existence of the OHF [19]. In other words, higher internal engagement is likely to attract more potential new users to it, and also makes it appealing in the eyes of those potential users. This leads us to the first research question (RQ1): Is there a causal relationship between engagement and the reach of an OHF?

Lurkers make up the majority of the user base of any online community. There are multiple reasons behind this phenomenon [20]. For example, lurkers might not find contents that would prompt them to speak up, breaking the silence of passive reading. Higher volumes of engaging content might drive the lurker to consume more, and eventually find something to respond to. More engaging content may also help new users, hesitant to contribute for an extended period of time, to get comfortable with the OHF norms and style of interaction to become an active contributor. Thus, we formulated the second research question (RQ2): Does delurking tend to occur at a higher rate when engagement increases?

OHFs must facilitate bonding between users in order to sustain voluntary participation. According to the common bond theory, users feel motivated to gel as a group because of bond-based personal attachment [7]. In other words, the more friends a user has, the more likely they are to return to the platform to contribute to it further. Therefore, it is of interest to find out whether engagement measurement can be used to inform (track) the development of virtual bonds in OHFs. To this end, we formulated our third research question (RQ3): Is the engagement value delivered by one forum user to another associated with the development of a personal bond (closer virtual relationship) between them?

One may also expect that those users who are successful in engaging their peers feel encouraged by the fact that they manage to help others and, hence, are more likely to keep contributing. To explore this relationship quantitatively, we formulated the fourth research question (RQ4): Does the success in engaging peers motivate a user to remain active in an OHF?

## Methods

### Engagement Metrics

**Figure 1:**
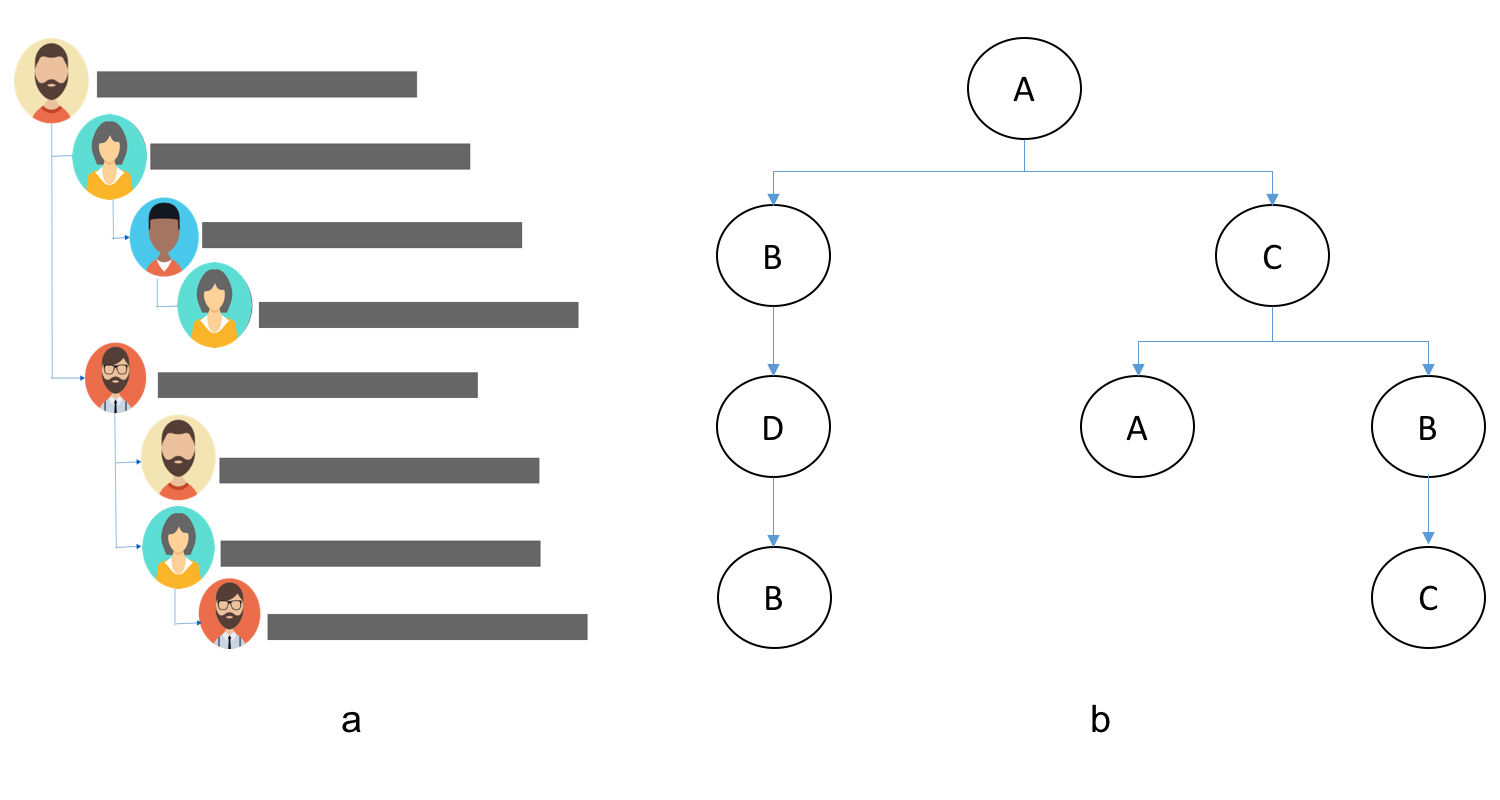
An online health forum communication thread: an illustrative example. (a) Possible structure of a communication thread, (b) represented as a network of directed relationships.

The value of an OHF is generated cooperatively by its users, as they communicate (ie, they author posts). In other words, each user deserves a credit for contributing to the overall value of an OHF (ie, the content it hosts, and consequently, the value it brings to the society). Figure 1 (part a) shows an example of the possible structure of a communication thread in an OHF. This thread can be represented as a network of directed relationships (see Figure 1, part b) reflecting which post “attracted” which. The metric engagement capacity is designed to quantify the credit that each user has earned for engaging their peers, taking into account the flow of communication between them. Note that this credit amount, if computed over a long time (over multiple threads), may also be indicative of the user’s ability to engage peers in the future.

The engagement capacity of a user is viewed as their share in the overall forum’s ability to attract posts, based on all the threads the user has contributed. It is assumed that engagement capacity can be positive only if a user’s content is responded to by other users in the forum. Thus, passive readers and users whose posts do not generate any response do not add to the engagement value of the forum.

To calculate the engagement capacity for each user, all of the engaging *subthreads* in a given OHF must be identified. Every path in the communication network (as in Figure 1, part b) that begins at the root in such a directed graph is called a subthread. Each post, submitted in response to any prior post in a subthread, increments the OHF’s overall engagement value by 1 unit. This 1 unit is shared by all the users who participated in the subthread that brought this 1-unit increase (in other words, attracted this new post). For example, the engagement value of 1 generated due to the response of user B to the subthread ABDB is shared between the users A, B, and D. Note that user B in part *self-engages* in this case.

Further, just as OHF users differ by their needs (eg, in seeking information vs emotional support [21,22]), some users may be more successful in engaging a certain set of peers over others. The metric targeted engagement capacity takes this into account and quantifies the total credit allocated to user *i* for successfully engaging user *j*; note that engagement-based relationships are *directed* and nonsymmetric. To put this in a context, for example, for answering RQ3, one can set to check whether targeted engagement can predict the formation of personal connections between users: to this end, the targeted engagement values and the events corresponding to the instances of personal connection formation can be monitored over time.

### The Technical Side of Engagement Quantification

To explain the exact formula for the engagement capacity computation, observe how the thread in Figure 1 has been developed. User A is the creator of the thread. First, user A gets 1 unit of engagement value for each of the direct responses of users B and C to this post. Further, when user D responds to user B’s post, the generated unit of engagement is shared between users A and B. Note that user C’s engagement value is not affected by D’s response, as C is not a part of the subthread to which D is responding. The exact share of each subthread contributor in a newly earned unit of engagement value is derived relying on the logic of cooperative game theory [23-25], according to which the OHF users can be viewed as playing games on *k*-coalitions, which are connected ordered sequences of player *appearances* [17].

**Figure 2:**
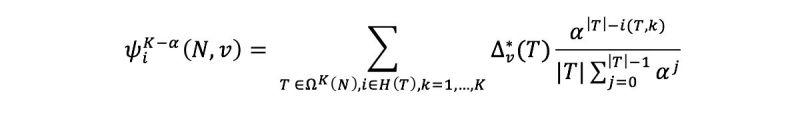
Engagement capacity equation (see text for explanation).

In the context of a forum of *N* users with *P* subthreads, let Ω^K^(*N*) represent the set of all *k*-coalitions, in which any player appears at most *K* times, and let *H* (*T*) represent the set of players in coalition *T.* To denote the position of player *i* appearing at most *K* times in a coalition, we use *i* (*T*, *k*), *k*=1,2,..., *K.* With the *value function* taken as a total number of post exchanges immediately succeeding such *engaging* subthreads *p is an element of P* that have the same membership, size, and structure as *k*-coalition *T is an element of* Ω^K^(*N*), the engagement capacity of user *I is an element of N* is computed by the equation in Figure 2.

The parameter α in this equation controls the distribution of credit for attracting a response among the users in the subthread that generates the response. With α=1, the formula gives equal credit to the authors of all the posts in the subthread. With α<1, the immediate predecessor of a newly submitted response gets more credit for attracting it, with the credit to the earlier predecessors discounted by the factors of α, α^2^, α^3^, etc, respectively. Note that this property of engagement capacity complies with an observation that the posts, newly attracted to a (long) forum thread, tend to respond to the latest prior contributions in this thread.

All the computations reported in this paper have been performed with α=0.8. With α=0.8, about half the credit for attracting a new post to a very long thread, to be shared by all the contributors, goes to the last 4 contributors, with the contributors further upthread getting less and less credit. A smaller value of α would reward the most recent contributors even more, to the point where the thread originator would get almost no credit. Note, however, that over all the contributions in a given thread, the thread’s originator is always guaranteed to earn more credit than any other contributor. Note also that the choice of α does not affect the computational effort required to perform the engagement capacity evaluation.

**Figure 3:**
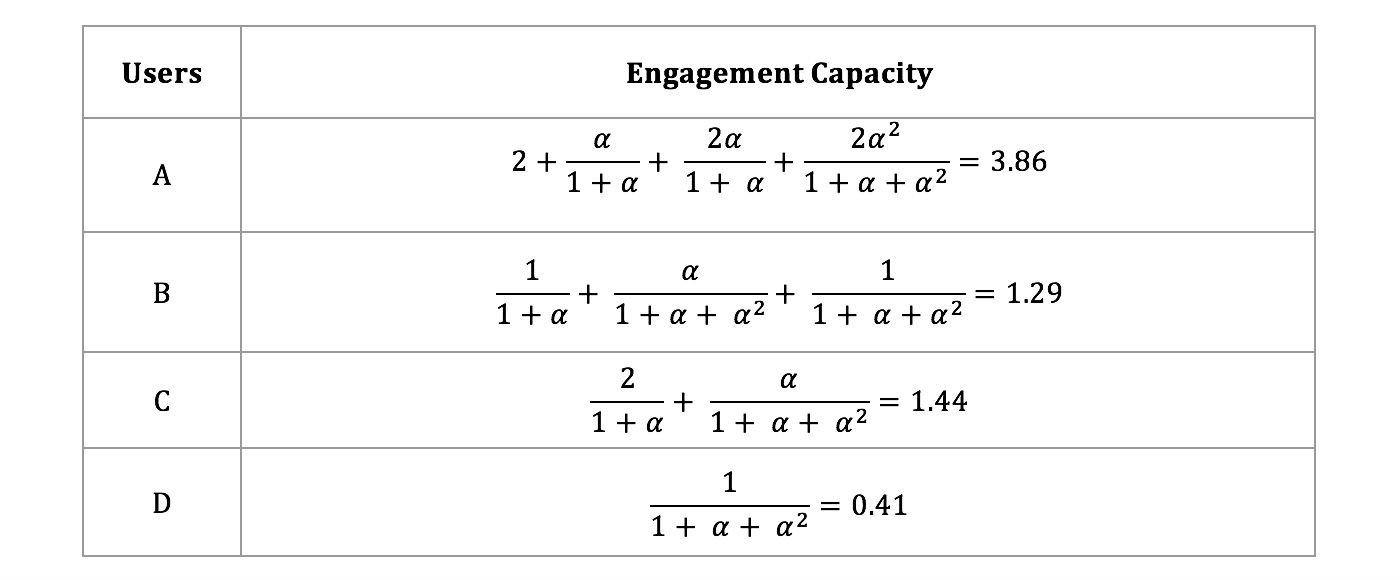
Engagement capacity values computed for the users in Figure 1.

To illustrate the application of the engagement capacity computation formula, observe that the thread in Figure 1 increments the forum’s engagement value by 7 units: 7 of its 8 posts have been submitted in response to some preceding post(s). Figure 3 reports how this value is distributed among the thread contributors.

Note that with each new piece of content (a new post or multiple posts) added to a forum in response to any prior post(s), the engagement analysis does not need to be redone for the past history of the forum. Each response to a prior post adds a value of 1, which is shared only by the users in a particular subthread, and hence, the engagement capacities of only those users need to be updated (incremented by a certain amount, per the engagement capacity computation formula). Hence, the runtime of an efficient engagement quantification algorithm is linear in the number of posts in a forum.

### Data Collection

We collected data from a big, active, and freely accessible online prohealth platform, MedHelp.org [26]. Each of its (approximately) 200 predominantly English-speaking OHFs is devoted to a specific health-related topic. The platform users interact through discussion boards, author personal journals, post notes, and post status updates on personal pages.

**Figure 4:**
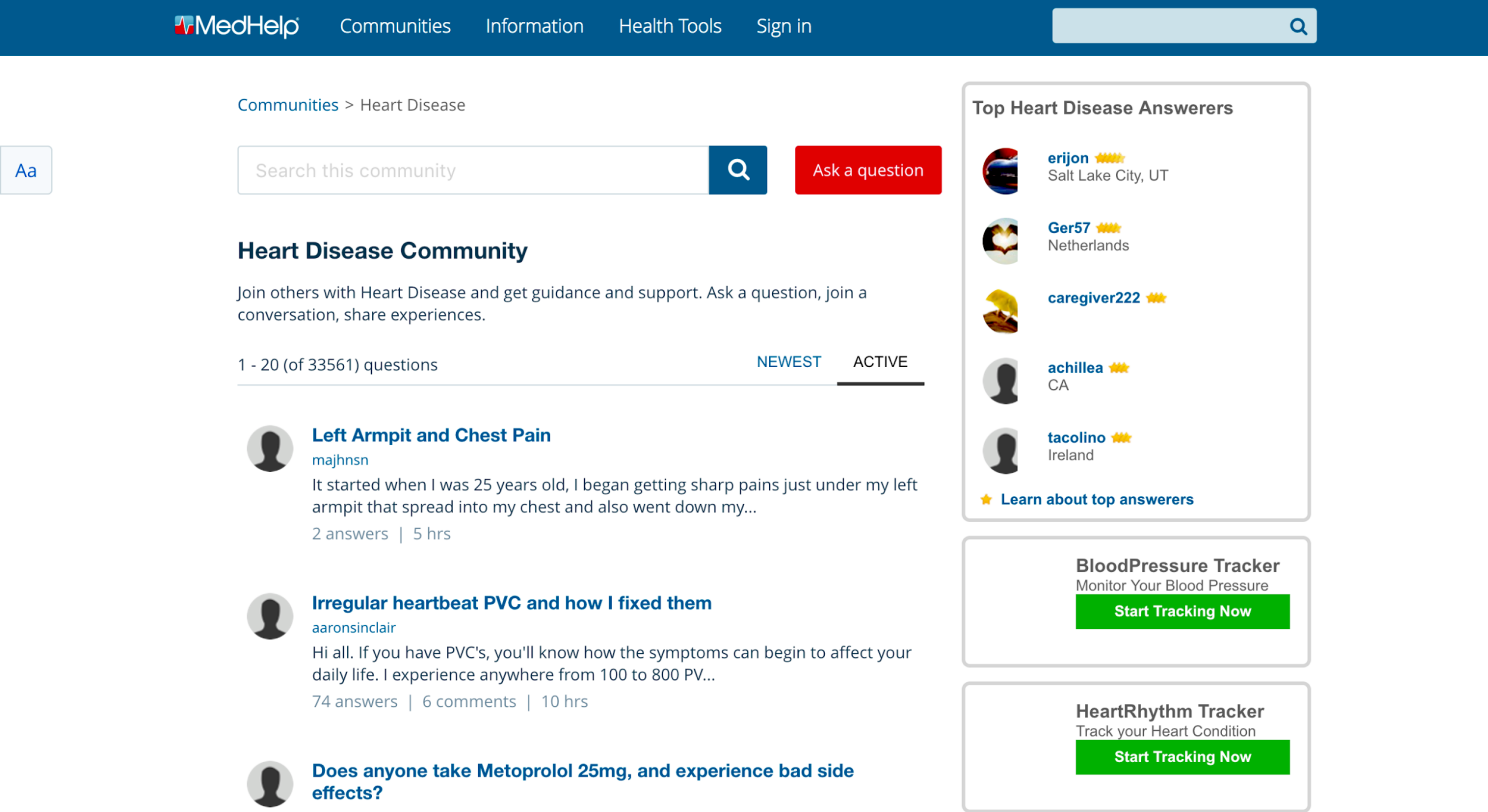
Screenshot of the Heart Disease community forum at MedHelp.org.

We collected the data from the Heart Disease, Diabetes, Substance Abuse, Fitness, Depression, Heart Rhythm, and Anxiety OHFs, all active between the years 1999 and 2016. The MedHelp.org data are publicly available [27]. In processing the data, we did not save users’ personal profile details, and we anonymized usernames. Figure 4 shows a random screenshot of the MedHelp Heart Disease community forum with snippets of several threads. There were approximately 200,000 threads, although some of the threads did not generate responses (see Table 1). We broke the timeline into fixed intervals for performing longitudinal analyses. Within each time interval, we tracked engagement capacity, targeted engagement capacity between users, number of newly registered users, and delurking and friendship-building events.

**Table 1:** Statistics (counts) for the observed online health forums (OHFs), rounded to thousands.

OHF community discussion topic	Total messages	Total threads	Threads of >1 posts	Contributing users
Heart Disease	104,000	32,000	23,000	31,000
Diabetes	14,000	4000	3000	5000
Substance Abuse	760,000	81,000	78,000	52,000
Fitness	19,000	5000	4000	9000
Depression	58,000	13,000	12,000	14,000
Heart Rhythm	90,000	18,000	17,000	15,000
Anxiety	166,000	34,000	30,000	33,000

### Data Analyses

We carried out the following procedures to answer RQ1-RQ4. For RQ1, we calculated the engagement values for each day over 17 years based on the observed communication on the platform (all 7 OHFs lumped together). In this calculation, each new post, generated *in response to* any prior post, contributed an engagement value of 1 to the overall *engagement value*. To quantify the *reach*, we recorded the number of new users joining the platform in each day. Answering RQ2 required the tracking of delurking—the phenomenon (event) where a user breaks the habit of passive reading by adding content to a forum, thereby extending its reach [28]. The OHFs examined in this study did not require their visitors to register for passive reading; therefore, for RQ2, we took delurking to occur whenever a registered user contributed a post after at least a month of inactivity. For RQ3, we assumed a personal virtual connection to have formed (been initiated) when a user posted a note on a peer’s profile front page for the first time. We calculated targeted engagement capacity values for user *i* engaging peer *j* for all user pairs (*i*, *j*). A binary indicator function was used to track the count of personal (virtual) connections resulting from user interaction in an open forum; the function took the value of 1 whenever user *j* posted a note on *i’s* wall. To answer RQ4, we computed the engagement capacity for each user over fixed time periods (1 week long). A binary indicator function was used to track whether the user was active (ie, contributed to the forum) in the following week; for example, the engagement value for user *i* was computed for week *t* and an indicator function was used to record whether user *i* contributed to the forum through posts or comments in week *t* +1.

### Granger Causality Test

We investigated RQ1 and RQ2 using Granger causality testing, a widely used tool for the analysis of joint temporal dynamics of multiple observed quantities (here, engagement value, new user count, and delurking). Per the theory of Granger causality, one signal (*X*_1_) is said to *Granger-cause* another signal (*X*_2_) if the past value(s) of *X*_1_ contains information that can predict *X*_2_ better than the information contained in the past value(s) of *X*_2_ would do alone [29]. In general, detecting a 1-way Granger causation is desired to definitively establish the nature of a cause-and-effect relationship between 2 temporally varying quantities. The 2-way Granger causality test is typically conducted to first detect any cause-and-effect relationship in both directions. If one observes that *X*_2_ Granger-causes *X*_1_, while *X*_1_ Granger-causes *X*_2_, then further analysis is done to determine the direction in which the causal effect is the strongest. In this study, we took the OHF engagement value as *X*_1_, and new user count and delurking rate as *X*_2_.

## Results

### Causal Relationship Between Engagement and Reach

**Figure 5:**
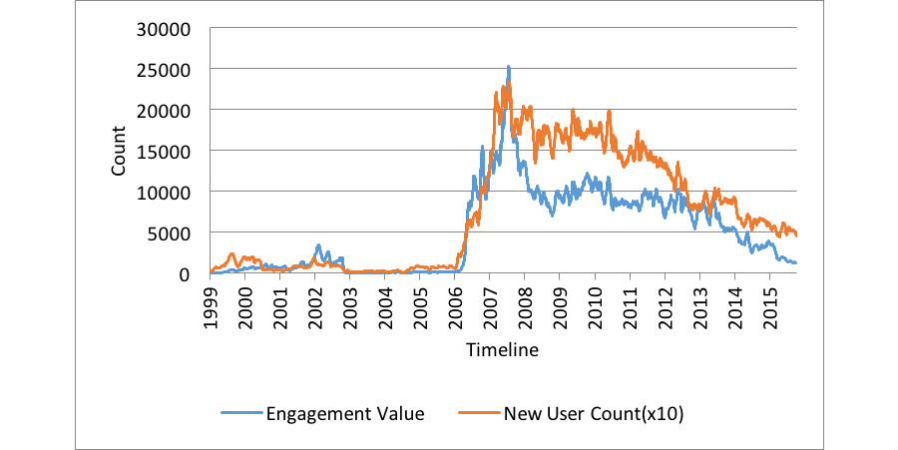
Temporal dynamics of the platform’s engagement value and new user counts (with new user counts scaled up by a factor of 10).

The Granger causality test performed between the platform’s engagement and new user count indicated that engagement Granger-caused reach (*P*=.02 with lag 2). The new user count, shown by the orange line in Figure 5, was incremented by 1 if a new user joined the forum; meanwhile, the engagement value of the platform, shown by the blue line, was incremented by 1 if a post was responded to. We calculated both these values for each day between 1999 and 2015. On average, 5076 posts on the platform were responded to daily, with 25,260 being the maximum number of responses. Note that we refer to all 7 OHFs combined as the “platform.”

**Figure 6:**
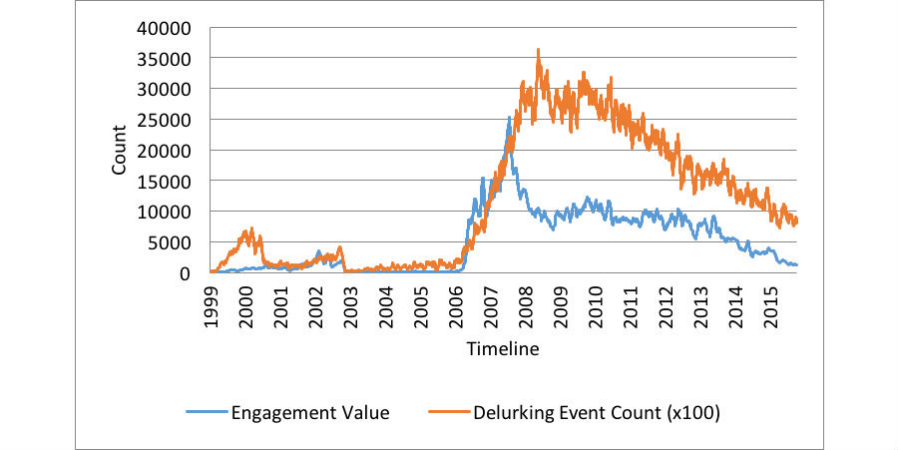
Temporal dynamics of the platform’s engagement value and delurking event counts (with delurking event counts scaled up by a factor of 100).

**Figure 7:**
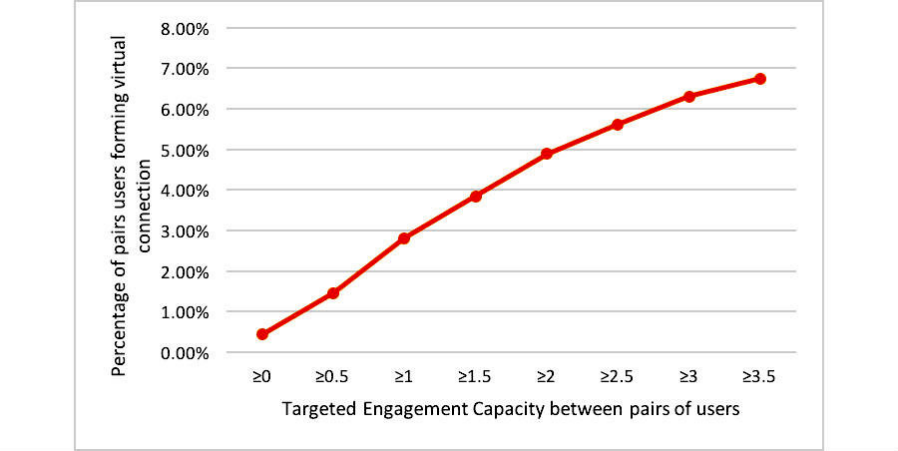
Proportion of total user pairs building a personal connection as a function of targeted engagement capacity between online health forum users.

### Causal Relationship Between Engagement and Delurking

Figure 6 shows that the fluctuations in the rate of delurking follow the fluctuations in engagement value. Granger causality analysis revealed a 1-way causation, with the hypothesis of engagement Granger-causing delurking supported by a significance of *P*<.001 with lag 2, and the hypothesis of delurking Granger-causing engagement supported by a significance *P*=.01 with lag 2. We thus concluded that we had a stronger support of the claim that engagement caused delurking (than the other way around). The count of the delurking events, shown by the orange line in Figure 6, was incremented by 1 if a registered user contributed content in 1 of the 7 forums after at least a month of inactivity. On average, 117 people responded to posts in a day after at least a month of inactivity (maximum 364 people per day).

### Targeted Engagement Capacity as a Predictor of the Development of Personal Connections Between Users

Figure 7 shows the relationship observed between the propensity of building personal (virtual) connections among OHF user pairs and the targeted engagement capacity delivered by 1 user in a pair to another user in this pair. Any 2 users have zero value if they have not responded to each other’s post in any of the 7 forums. Also, note that targeted engagement is directed and nonsymmetric; that is, user *i* engaging user *j* is not the same as *j* engaging *i*. We calculated this metric over 1621,635 pairs and observed a total of 8482 notes in the OHFs. The percentage of pairs of users forming personal virtual connections out of 1,621,635 is shown on the vertical axis. For example, the point (x=1.5, y=3.98%) indicates that, out of 1,621,635 pairs of people accounted for in the targeted engagement calculation, approximately 3.98% ended up developing personal virtual connections if 1 user in the pair managed to engage the other user to earn a targeted engagement value of at least 1.5. Overall, the more frequently user *i* managed to engage user *j* (prompting *j* to respond), the more likely *i* was to receive a posted personal note from *j* for the first time. The mean value of targeted engagement capacity for all the users with established personal connections is 1.6 (SD 4.8). A regression line, fit to the curve in Figure 7, has an adjusted *R*^2^=.97.

### Engagement Capacity as a Predictor of Future Activity

**Figure 8:**
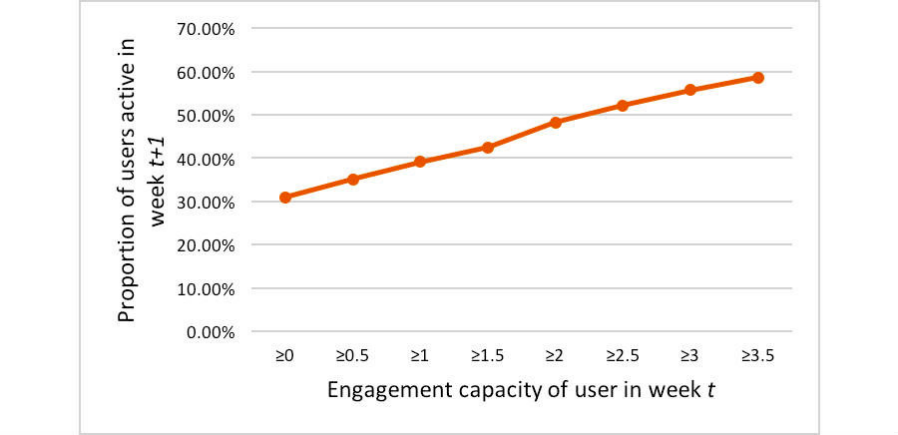
Propensity of users to stay active in an online health forum as a function of their engagement capacity earned over time.

Figure 8 shows the relationship observed between the propensity of a user to stay active in an OHF and the engagement capacity of this user earned over the past week. We calculated the engagement capacity of each user for each week. For example, the point (x=1, y=39%) in Figure 8 indicates that approximately 39% of users in the platform tended to stay active in the week *t+1* if their posts earned them the engagement capacity of at least 1 in week *t*. Users who received high engagement for their posts were more likely to contribute to the forum. The vertical axis in Figure 8 shows the proportion of users active in week *t* +1 with respect to their engagement capacity in week *t*. We considered a user to be active if they participated by posting content in any of the forum threads in week *t* +1. A regression line, fit to the curve in Figure 8, has an adjusted *R*^2^=.99.

## Discussion

### Principal Findings

The 7 OHFs we observed in this study were diverse, with a varied number of posts and users. Some of the users were simultaneously active in more than 1 OHF. Granger causality testing performed on observational data revealed that engagement Granger-caused reach of the platform. The analysis of delurking showed a 2-way relationship between engagement and delurking: this reveals an interdependence of these 2 variables; further investigation indicated that changes in engagement value dynamics tended to precede those in the delurking count. In summary, engagement measurement appears to provide useful information about the retention rate of users in the platform. We also found engagement capacity to be an informative metric for predicting the propensity of a user to contribute to an OHF. Further, targeted engagement, as the measure of the amount of directed communication between a pair of users, provided information about the development of a virtual connection between them.

### Practical Implications

Targeted engagement computation can be useful for predicting the retention rate of users in the platform, which is in line with the common bond theory. Specifically, in the future, this metric can become an integral component of OHF thread recommender systems [16], where suitable threads can be recommended to those users who are more likely to develop personal connections through communication. As noted in the Introduction section, previously proposed metrics of OHF activity, including those that rely on social network analysis tools, have been predominantly used for descriptive purposes. Our results suggest that the engagement capacity and targeted engagement capacity metrics can be used for predictive, and perhaps prescriptive, purposes. Indeed, engagement measurement allows OHF managers to move beyond merely counting contributions, “likes,” and time spent by users online, toward identifying such posts and users that are inherently engaging. To inform managerial insights, we foresee outputs based on engagement measurement being displayed in a graphical form, such as plots, also enabling time-dependent tracking. Downward trends in engagement capacity of superusers, engagement value of the platform, etc, can be good indicators for managers to take proactive measures. As part of future developments in this direction, we anticipate an interest from the research community to work toward finding patterns in downward engagement trends and critical points for the platform’s growth.

Importantly, *why* or *how* those posts and users manage to be engaging can be studied with the help of text mining techniques [29]. The success of an OHF depends on user participation. To promote the growth of the user network, practitioners are typically interested in identifying those users who contribute most toward the development of the OHF user base as a community [30]. The most important, or influential, users of prohealth platforms are often referred to as core users or superusers [31]. These individuals are integral to OHF growth, as they willingly provide continuous support, advice, and information to other users. The engagement metric offers a new way of understanding what it means for a user to be “important” to an OHF, and the answers to RQ1-RQ4 help pave the way for the development of calculated interventions for strategically growing OHFs, informed by engagement measurement.

Previously, superusers (and their subtypes) have been defined in the literature [32-34]. These definitions, developed qualitatively, quantitatively, and graphically, have studied a variety of observed parameters, including participation style, social support type, thread initiation, participation inequality, and user life cycles. This study contributes to an enhancement of those existing definitions, using engagement metrics. Superusers, recognized through the lens of the engagement metric, can now be viewed as users having an innate ability to engage others. Indeed, while previous works have considered frequency of contributions and similar frequency-based measurements as the main metrics for identifying superusers, our work allows for use of a metric such as engagement per post, emphasizing the skill of being engaging as a key quality for a superuser to have.

**Figure 9:**
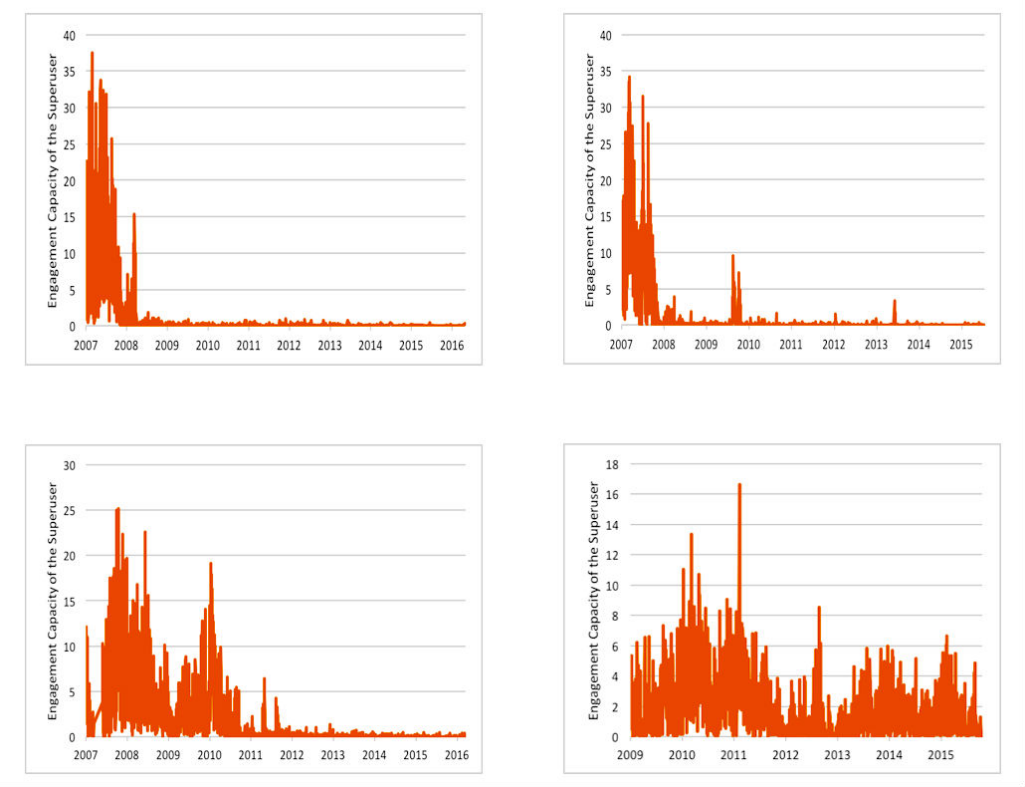
Engagement capacity dynamics of 4 of the top 10 most engaging online health forum users.

Displaying the calculated user engagement capacities, earned over time, to users can potentially increase their future contributions via gamification [35]. Further, highly engaging users can be asked or incentivized to spend more time on an OHF, as they now can be recognized as the individuals capable of helping the OHF succeed. Designing suitable incentives to encourage participation of such users will keep the platform more engaging by attracting more posts. Figure 9 shows the engagement capacity, per day, for 4 of the studied OHFs’ top 10 most engaging users. Interestingly, the plotted patterns reveal that certain of the most engaging users may have been highly active for prolonged time periods, while others may have been highly active only for shorter time periods. OHF managers can take proactive measures to motivate superusers when there is a downward trend in their engagement capacity. For example, the superuser in Figure 9 (top left) can be seen to have a sudden engagement drop in mid-2008. This could have been avoided by developing suitable interventions to help the superuser stay committed to the platform, or to make sure that other superusers “pick up the slack” in time.

Moreover, further research into the possible exact definitions of engagement-based superusers and quantification of a downward trend in their contribution is in order. For example, nominating a superuser can become time dependent, based on a user receiving a high engagement score, such as over a month of forum life. Also, normalized engagement capacity (ie, engagement capacity per post) can be used to make engagement measurement less subjective.

### Limitations

The growth of an online forum is dependent on various factors (eg, organizational, psychological, and sociological). Therefore, the engagement of an OHF should be considered as one of the many factors, and not the only factor, responsible for causing the OHF to grow.

On the technical side, the Granger causality test output is known to depend significantly on the functional form of regression used to test a relationship between the variables [36,37]. Further, one must be aware that unobservable variables may play a role in the results of such test, and hence, must be careful not to overinterpret even statistically significant conclusions.

Also, engagement increases as more users join the platform and respond to contributed content; however, conflicts might emerge when online communities pass a certain threshold. While this issue might not be acute in OHF communication, as OHFs are typically well moderated, the nature of engagement—desirable or undesirable—has to be taken into account during the generation of insights based on engagement analyses.

### Conclusion

Our study shows that engagement is one of the important factors responsible for growth of OHFs; the theory-supported engagement metric provides a framework for systematically assessing a platform’s health by quantifying users’ innate ability to engage others. The predictive property of this metric can potentially inform the development of incentive schemes such as badges and monetary rewards, thread recommender systems, and identification of superusers. Using metrics such as frequency of contribution might result in a causation or correlation issue in identifying superusers, as it favors existing users as opposed to new users joining the platform. By tracking the engagement per post metric, forum managers can overcome this issue, in that users’ innate ability to engage their peers can be identified from the time they start contributing to the platform. Research can now be done toward answering the question “what makes a user engaging?”

## Abbreviations

OHF: online health forum
RQ: research question
